# Transcriptomic Analysis of *Mycobacterium leprae-*Stimulated Response in Peripheral Blood Mononuclear Cells Reveal Potential Biomarkers for Early Diagnosis of Leprosy

**DOI:** 10.3389/fcimb.2021.714396

**Published:** 2021-12-21

**Authors:** You-Hua Yuan, Jian Liu, Yuan-Gang You, Xiao-Hua Chen, Lian-Chao Yuan, Yan Wen, Huan Ying Li, Ying Zhang

**Affiliations:** ^1^ Department of Clinical Microbiology, Henan Provincial People’s Hospital, People’s Hospital of Zhengzhou University and People’s Hospital of Henan University, Zhengzhou, China; ^2^ Beijing Tropical Medicine Research Institute, Beijing Friendship Hospital, Capital Medical University, Beijing, China; ^3^ Beijing Key Laboratory for Research on Prevention and Treatment of Tropical Diseases, Capital Medical University, Beijing, China; ^4^ State Key Laboratory for the Diagnosis and Treatment of Infectious Diseases, Collaborative Innovation Center for Diagnosis and Treatment of Infectious Diseases, The First Affiliated Hospital, Zhejiang University School of Medicine, Hangzhou, China

**Keywords:** *Mycobacterium leprae*, leprosy, biomarkers, diagnosis, transcriptome analysis

## Abstract

We aimed to identify an unique host transcriptional signature in peripheral blood mononuclear cells (PBMCs) in response to *Mycobacterium leprae* antigens to distinguish between patients with leprosy and non-leprosy controls for early diagnosis of the disease. Sixteen individuals were enrolled in the discovery cohort [eight patients with leprosy, comprising four multibacillary (MB) and four paucibacillary (PB); and eight non-leprosy controls, comprising four healthy house contacts (HHCs) and four endemic controls (ECs)]. The differences in the transcriptome response of PBMCs to *M. leprae* sonicate antigen were evaluated between leprosy patients and non-leprosy controls, and 12 differentially expressed genes (*CCL2/MCP-1*, *IL-8*, *JAKM*, *ATP, ND1*, *SERP*, *FLJ10489*, *LINC00659*, *LOC34487*, *LOC101928143*, *MIR22*, and *NCF1C*) were identified. The accuracy of the 12 differentially expressed genes was further validated for the diagnosis of leprosy using real-time quantitative PCR in 82 individuals (13 MB, 10 PB, 37 HHCs, and 22 ECs) in the validation cohort. We found that a 5 gene signature set *IL-8*, *CCL2/MCP-1*, *SERP*, *LINC00659* and *FLJ10489* had a suitable performance in discriminating leprosy from ECs. In addition, elevated expression of *IL-8*, *CCL2/MCP-1*, *SERP* and *LINC00659* was associated with MB diagnosis compared with ECs, whereas increased expression of *IL-8*, *CCL2/MCP-1*, *SERP* and *FLJ10489* was found to be useful biomarkers for PB diagnosis from ECs. Moreover, we found decreased expression of *NCF1C* among leprosy patients could distinguish leprosy from HHCs, whereas higher expression of *CCL2* among MB than PB could distinguish different leprosy patients. In conclusion, among the 12 candidate host genes identified, a three gene signature *IL-8, CCL2/MCP-1*, and *SERP* showed the best performance in distinguishing leprosy patients from healthy controls. These findings may have implications for developing a rapid blood-based test for early diagnosis of leprosy.

## Introduction

Leprosy is a chronic infectious disease caused by *Mycobacterium leprae*. Although curable by multidrug therapy, leprosy remains a significant health problem in South-East Asia (e.g., India), North and Central Africa (e.g., Central African Republic and Democratic Republic of the Congo), Oceania (e.g., Indonesia and Papua New Guinea), and the Americas (e.g., Brazil and Mexico) (World Health Organization, 2017). According to the World Health Organization (WHO) in 2020, of more than 230,000 new cases with leprosy, 10,816 were detected in people with grade-2 disabilities globally (https://www.who.int/health-topics/leprosy) (World Health Organization, 2021). The early diagnosis of leprosy leads to breaking the chain of transmission and reducing the number of grade-2 disability cases in a given community. However, an accurate diagnosis of leprosy is still a challenge. Skin slit smear acid-fast staining is rapid and economic but has very low sensitivity and specificity (Mohanty et al., 2020). Definitive diagnosis of leprosy by clinic and pathological features requires experienced physicians. Although the host biomarkers with a positive immune response to *M. leprae* through whole-blood assay have potential diagnostic value for leprosy, especially for paucibacillary (PB) patients (Chen et al., 2019; Geluk et al., 2010; Hungria et al., 2017; Sampaio et al., 2011; van Hooij and Geluk, 2021), there is currently no diagnostic test based on transcriptomic analysis of leprosy patients in China. Therefore, the development of an accurate clinical diagnostic test is urgently needed.

Here, to generate a broad transcriptome profile that has potential as biomarkers for distinguishing different disease states, we used RNA sequencing (RNA-seq) to identify candidate host biomarkers that are differentially expressed in the *M. leprae-*stimulated peripheral blood mononuclear cells (PBMCs) of multibacillary (MB) and PB leprosy patients and non-leprosy controls, comprising healthy house contacts (HHCs) and endemic controls (ECs). The differentially expressed genes (DEGs) identified in this study can serve as useful biomarkers and provide a good foundation for the development of a useful blood test for rapid diagnosis of leprosy.

## Materials and Methods

### Ethics Statement

This study was approved by the Medical Ethics Committee of Beijing Friendship Hospital, Capital Medical University, Beijing, P.R. China. Written informed consent was obtained from all adult participants. All the procedures that involved human participants were performed in accordance with the ethical standards of the institutional and/or national research committee and Declaration of Helsinki, 1964, and its later amendments or comparable ethical standards.

### Human Subjects

We recruited eight leprosy patients (four each of MB and PB) and eight non-leprosy controls (four each of HHCs and ECs) from the Honghe Autonomous Prefecture in the Yunnan Province, Southwest China, during October 2014, as the exploration cohort. All of the individuals in this study were of the same ethnic group. Among the recruited patients, three were males and five were females. Then, a validation cohort of 82 individuals (13 MB, 10 PB, 37 HHCs, and 22 ECs) was recruited in the same region from February 2015 to May 2016. Leprosy patients were classified using the Ridley–Jopling classification (Ridley and Jopling, 1966) and into two groups, MB and PB, according to the WHO operational classification (WHO Expert Committee on Leprosy, 1998; Reibel et al., 2015). We recorded the basic information and clinic characteristics of the leprosy patients. The median and interquartile range (IQR) of the treatment duration were 4 months (1–7 months) and 1 month (1–5 months) for MB and PB leprosy patients, respectively. Age at the time of leprosy diagnosis ranged from 21 to 59 years, with a median of 39 years. There were 10 males and 6 females in the exploration cohort and 45 males and 37 females in the validation cohort. HHCs had been living in the same house as the adult leprosy patient. ECs were within the normal controls who lived in the same community as the leprosy patients (**Table 1**).

**Table 1:** Clinical information of leprosy patients and controls.

Classification (n)		WHO^*^RJ(n)		Number of cases	Gender ratio	Age (years)	Ethnicity	Bacterial index	Treatment duration (months)	
					(M/F)	(mean ± SD)	(Han/minority)	(BI)	Median	IQR
**Discovery cohort**	Leprosy patients	MB	BL	4	2/2	23.8 ± 10.6	1/3	1.16–4+	4	1–7
		PB	BT	4	4/0	43.8 ± 13.6	1/3	0	1	1–5
	Controls	HHC		4	2/2	37.5 ± 2.9	2/2	/	/	/
		EC		4	2/2	43.8 ± 10.5	4/0	/	/	/
**Validation cohort**	Leprosy patients	MB	BL	13	9/4	42.0 ± 11.1	4/9	0.8–5	5.5	1–7
		PB	BT	8	4/4	39.0 ± 19.2	2/6	1+	3.5	1–7
			TT	2	2/0	27.0 ± 18.3	2/0	0		
	Controls	HHC		37	20/17	33.1 ± 13.7	28/9	/	/	/
		EC		22	10/12	34.6 ± 12.8	17/5	/	/	/

^*^WHO: Operational classification proposed by the World Health Organization.

n, number of patients; RJ, Ridley–Jopling classification; MB, multibacillary; PB, paucibacillary; HHC, healthy house contact; EC, endemic control; BL, borderline lepromatous; BT, borderline tuberculoid; TT, tuberculoid tuberculoid; IQR, interquartile range.

### Preparation of *M. leprae* Sonicate

*M. leprae* whole-cell sonicate was obtained through the NIH/NIAID Leprosy Contract N01-AI-25469 at Colorado State University. Inactivated (irradiated) armadillo-derived *M. leprae* whole cells were probe sonicated using a Sanyo sonicator (Misonix, Cat. No. S4000, USA) to >95% breakage to produce a whole-cell sonicate (Cat. No. NR-19329, NIH, USA).

### Blood Samples and PBMC Acquisition

Peripheral blood (15 mL) was placed into EDTA tubes. PBMCs were isolated from peripheral blood using Ficoll-Paque separation (CEDARLANE, Cat. No. CL5020). Briefly, peripheral blood was centrifuged at 3,000 × *g* for 10 min to obtain a buffy coat. The supernatant containing plasma was removed and the buffy coat was diluted with an equal volume of RPMI-1640 (Invitrogen-GIBCO; Invitrogen). A total of 8 mL of the diluted buffy coat was layered over 4 mL of Ficoll-Paque (CEDARLANE). Gradients were centrifuged at 3,000 × *g* for 20 min at 20–30°C in a swinging-bucket rotor without an applied brake. The PBMC interface was carefully removed by pipetting and washing with RPMI-1640 and centrifuging at 2000 × *g* for 5 min. PBMC pellets were suspended in 5 mL of cold red blood cell lysis buffer (Sigma-Aldrich) and incubated for 10 min at room temperature with gentle mixing to lyse the contaminating red blood cells, followed by a wash with RPMI-1640. Cell number and viability were determined using a Countess Automated Cell Counter (Invitrogen). Non-viable cells were identified by staining with trypan blue, and cell viability was calculated using the total cell count of non- and viable cells. The gradients were centrifuged to obtain the mononuclear cell fraction, which was aspirated and washed twice with RPMI-1640. PBMCs were counted on a haemocytometer and were adjusted to a concentration of 2 × 10^6^ cells per mL in RPMI-1640. Then, the cells were divided into aliquots (990 µL per well of 12-well plate) and 10 µL *M. leprae* whole-cell sonicate antigen (1 mg/mL) was added to each well, followed by incubation for 12 h at 37°C.

### Total RNA Extraction and RNA-seq

Total RNA was extracted by using the TRIzol-mediated isolation protocol (Life Technologies, Grand Island, NY) following the manufacturer’s instructions as previously described (Yuan et al., 2017). To determine the concentration of RNA, the absorbance at 260 nm was measured using a Nanodrop one spectrometer (Thermo Fisher Scientific Inc., Waltham, MA). The RNA integrity number (RIN) and 28S:18S ratio were also measured, and total RNA samples with >10 mg, RIN > 7.0, and a 28S:18S ratio > 1.8 were used in subsequent experiments.

### cDNA Library Preparation and Sequencing

The construction of the libraries and RNA-seq were performed at Shanghai Novel Bioinformatics Company (Shanghai, China). The poly (A) messenger RNA (mRNA) purification, mRNA fragmentation, and the cDNA library preparation for transcriptome sequencing were conducted using SYBR Premix Ex Taq™ II Kit (Perfect Real Time; Takara Bio, Cat. No. RR820A) and PrimeScript RT reagent Kit (Takara Bio, Cat. No. RR0377A). Then, the paired-end cDNA library with an insert size of 150 bp was prepared following the protocols proposed by Illumina. The cDNA libraries were sequenced on the Illumina HiSeq 2000 genomic sequencer platform at Shanghai Novel Bioinformatics Company.

### RNA-seq Data Analysis

The differentially expressed genes (DEGs) were analyzed between samples using the DESeq algorithm (Anders et al., 2010). Then a p-value was obtained, which was corrected using the false discovery rate method (Benjamini Y, et al., 2001). Parameters for classifying significant DEGs are ≥2-fold differences (|log_2_FC| ≥ 1; FC: fold change of expression) and ≥5,000 raw reads in the transcript abundance as well as p < 0.05.

By searching the National Center for Biotechnology Information (NCBI), UniProt, GO (gene ontology), and KEGG database (https://www.who.int/health-topics/leprosy), and BLAST (Basic Local Alignment Search Tool) alignment were performed to determine the functional annotation of DEGs. The best matches were selected to annotate the DEGs. Finally, DEGs were subjected to GO and KEGG functional analysis, utilizing default parameters, to annotate the DEGs’ major GO and KEGG categories.

### Verification of RNA-seq Data Using Reverse Transcription Quantitative Polymerase Chain Reaction

To validate the RNA-seq data, RT-qPCR was performed using SYBR Premix Ex Taq™ II Kit (Perfect Real Time). The 16 RNA samples used in the discovery phase were also used in the RNA-seq analysis of the verification. The primers were selected and analysed using the Primer Premier Software (version 5.0) (**Supplementary Table S1**). The reaction mix contained 6.25 μL of SYBR Premix Ex Taq™ II (2×), 0.25 μL of ROX Reference Dye II (50×), 1 μL of 10 μM primer mix, 1 μL of cDNA, and water to complete a final volume of 12.5 μL. Cycling conditions were: 95 °C for 30 s, 40 cycles at 95 °C for 5 s, and 60 °C for 34 s. All RT-qPCR experiments were performed using three biological and three technical replicates on an Applied Biosystems 7500 Fast real-time PCR system (Applied Biosystems). *GAPDH* mRNA was used as an internal control, and the 2^−ΔΔCT^ method was used to calculate the fold change (where ΔCt = Ct mRNA – Ct *GAPDH*, and ΔΔCt = ΔCt stimulated − ΔCt unstimulated).

### Statistical Analysis

Statistical analysis was performed mainly using the GraphPad Prism software version 8.0 (GraphPad Software Inc., San Diego, CA, USA) and SPSS 25.0. The nonparametric Mann–Whitney U-test was used to analyze differences between the two groups. A p-value < 0.05 was considered significant.

## Results

### Study Design Overview and Basic Characteristics Of Participants

**Figure 1:**
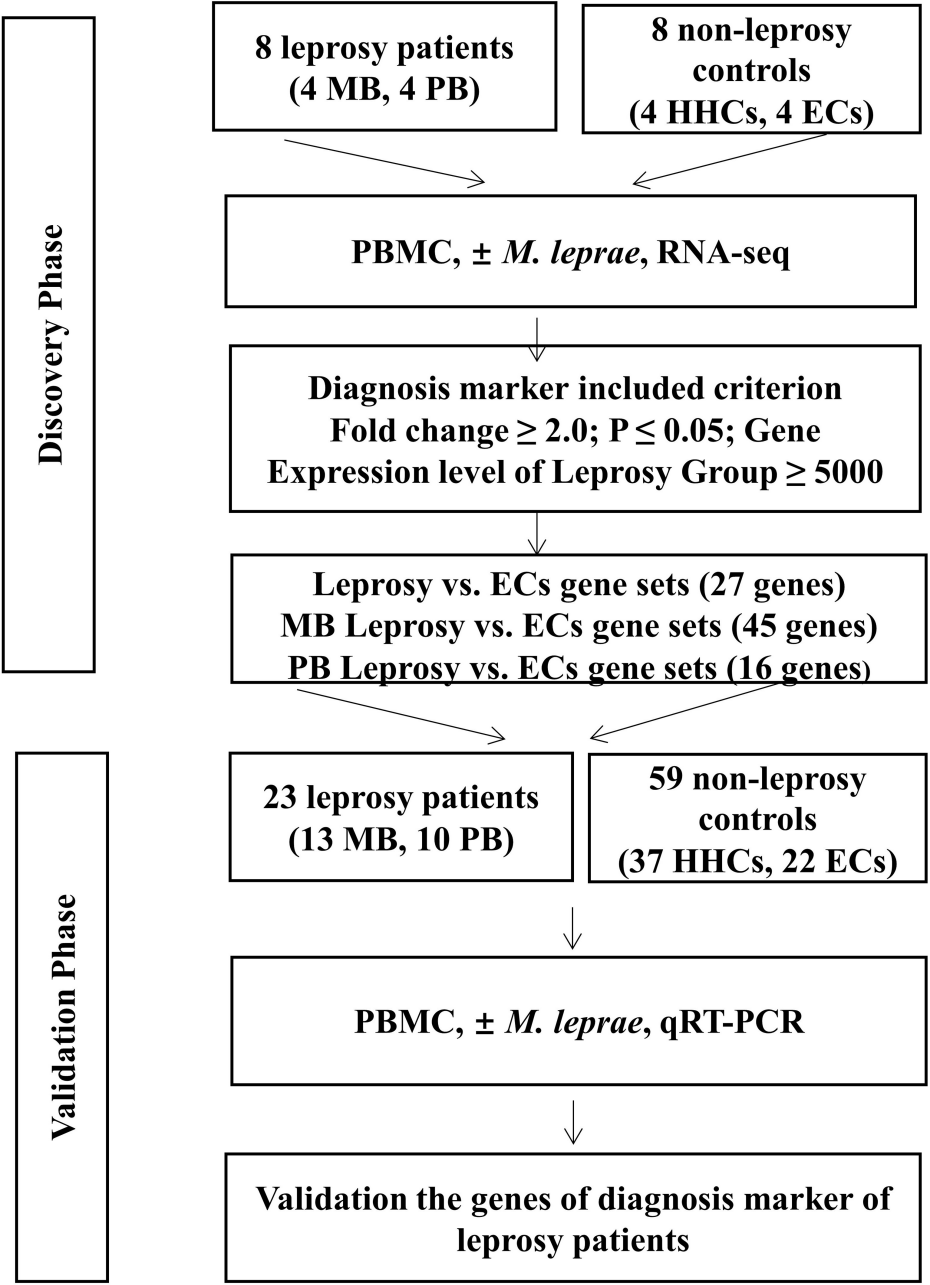
Workflow of the study. The study was subdivided into the discovery and validation phases. In the discovery phase, we enrolled eight leprosy patients (four each of MB and PB) and eight non-leprosy controls (four each of HHCs and ECs). PBMCs of all subjects were subjected to *M. leprae* sonicate stimulation and the genes with a fold change (FC) ≥2(P < 0.05) in expression levels of the patient group ≥5,000 raw reads in RNA-seq analysis were identified. From this set of genes, we derived a set of genes as potential diagnostic biomarkers for MB and PB patients. In the validation phase, we enrolled 23 leprosy patients (13 MB and 10 PB) and 59 non-leprosy controls (37 HHCs and 22 ECs) and obtained RNA from *M. leprae-*stimulated PBMCs. We validated the genes as diagnostic biomarkers of leprosy using qRT-PCR. MB, multibacillary; PB, paucibacillary; HHCs, healthy house contacts; ECs, endemic controls; PBMC, peripheral blood mononuclear cells.

Our study followed a two-step design (**Figure 1**). First, we enrolled a discovery cohort of eight leprosy patients (four each of MB and PB) and eight non-leprosy controls (four each of HHCs and ECs). By comparing *M. leprae* antigen-stimulated gene expression in PBMCs from whole blood between the two groups (i.e., leprosy patients and non-leprosy controls) using RNA-seq, we derived a gene set that was differentially expressed among the leprosy patient and control groups. Next, we employed the upregulated genes that were differentially induced in a validation cohort of 23 leprosy patients (13 MB and 10 PB) and 59 non-leprosy controls (37 HHCs and 22 ECs), and obtained RNA from the PBMCs from whole blood stimulated with *M. leprae* antigens. The selected genes were validated as the diagnostic biomarkers of leprosy using RT-qPCR (**Figure 1**).

Demographic and clinical characteristics of the cohorts are listed in **Table 1**. The median and IQR of the treatment duration were 4 months (1–7 months) and 1 month (1–5 months) for MB and PB leprosy patients of the exploration cohort, respectively. The median and IQR of the treatment duration were 5.5 months (1–7 months) and 3.5 months (1–7 months) for MB and PB leprosy patients of the validation cohort, respectively.

### Transcriptomic Differences in *M. leprae* Antigen-Stimulated PBMCs Between Leprosy Patients and Controls by RNA-seq

**Figure 2:**
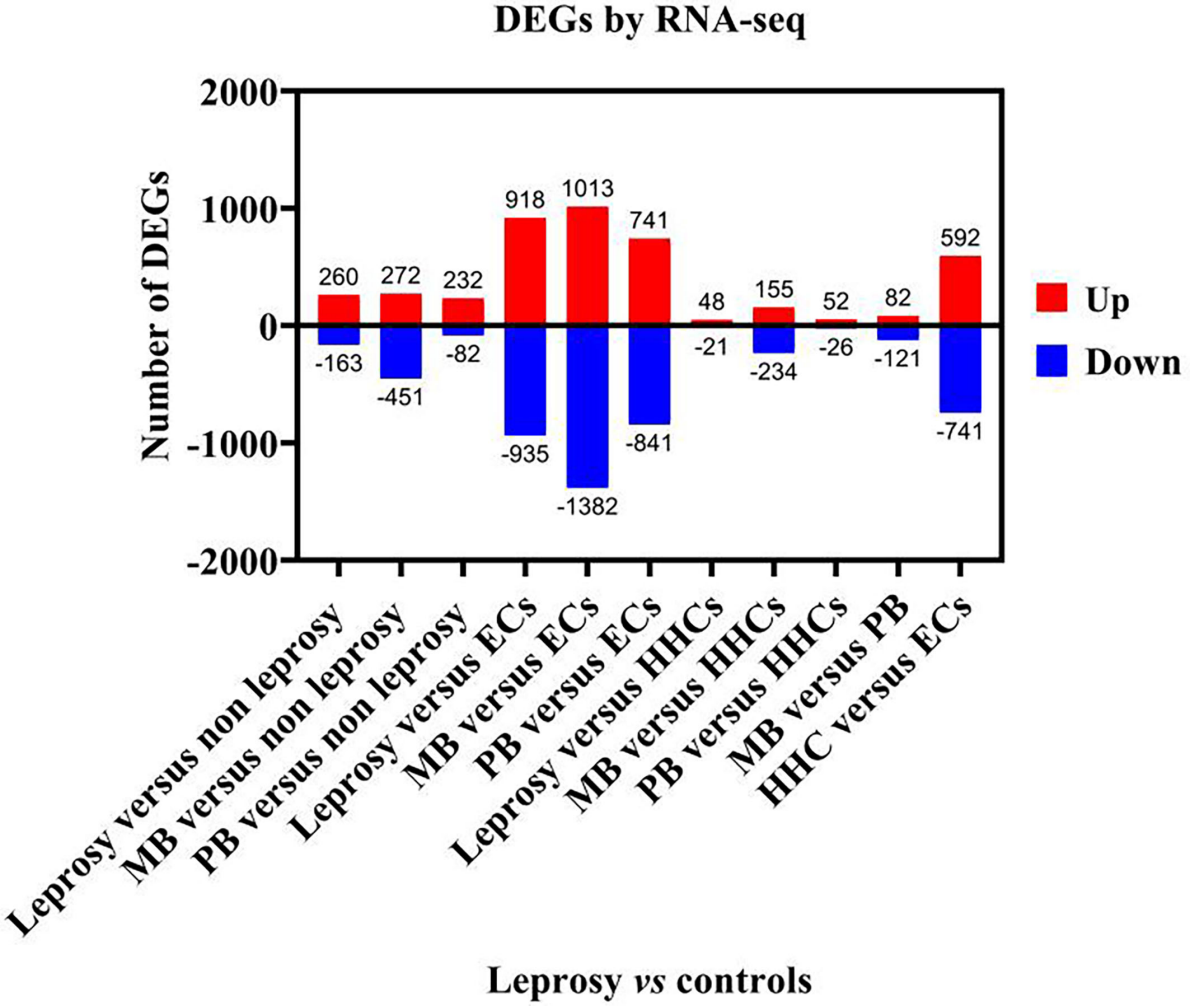
The number of deferentially expressed transcripts altered in leprosy (MB + PB) patients compared to non-leprosy controls. DEGs, differentially expressed genes; RNA-seq, RNA sequencing; MB, multibacillary; PB, paucibacillary; HHCs, healthy house contacts; ECs, endemic controls.

We analysed the results of DEGs by RNA-seq of 16 samples (**Figure 2**). When the transcriptome profiles of the leprosy patients were compared to those of the non-leprosy controls, a total of 423 DEGs were obtained, with 260 and 163 DEGs being upregulated and downregulated, respectively. In addition, 723 and 314 DEGs were obtained from MB and PB leprosy patients, with 272 and 232 DEGs being upregulated and 451 and 82 DEGs downregulated in MB and PB leprosy patients compared to non-leprosy controls, respectively (**Figure 2**).

**Figure 3:**
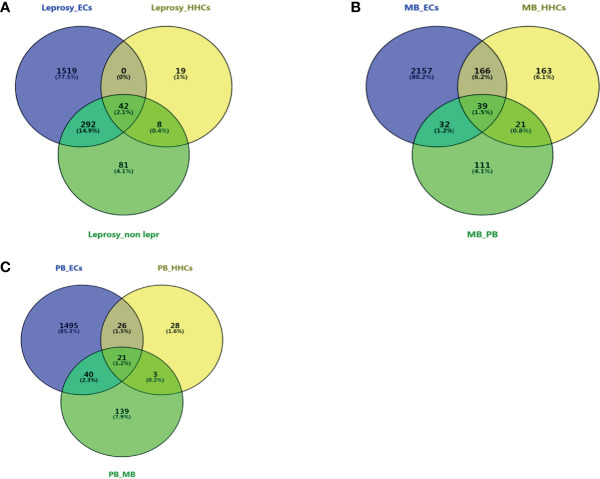
Venn diagram of DEGs at different groups of leprosy patients and controls. Three-way Venn diagrams of genes significantly and differentially expressed in leprosy patients and non-leprosy controls. **(A)** Venn diagram of DEGs between leprosy patients and ECs, HHCs, and non-leprosy controls. **(B)** Venn diagram of DEGs between MB leprosy patients and ECs, HHCs, and PB leprosy patients. **(C)** Venn diagram of DEGs between PB leprosy patients and ECs, HHCs, and MB leprosy patients. MB, multibacillary; PB, paucibacillary; HHCs, healthy house contacts; ECs, endemic controls; DEG, differentially expressed gene.

In order to identify the common DEGs between leprosy patients and healthy controls, Venn diagram analysis was used to compare the transcriptomic differences in *M. leprae* antigen-stimulated PBMCs of leprosy patients with those of the controls. The DEGs (p-value ≤ 0.05 after false discovery rate correction) were obtained between the leprosy and non-leprosy groups and described in a Venn diagram, where 42 of 1,961 DEGs showed significantly altered expression in the leprosy patients compared to the three control groups (ECs, HHCs, and non-leprosy controls [EC+HHCs]) (**Figure 3A**); 39 of 2,689 DEGs showed a remarkably altered expression in MB leprosy patients compared to the other three groups (ECs, HHCs, and PB; **Figure 3B**), and 21 of 1,752 DEGs showed a remarkably altered expression in PB leprosy patients compared to the other three groups (ECs, HHCs, and MB; **Figure 3C**).

### DEGs Between Leprosy Patients and Non-Leprosy Controls as Potential Diagnostic Biomarkers

**Figure 4:**
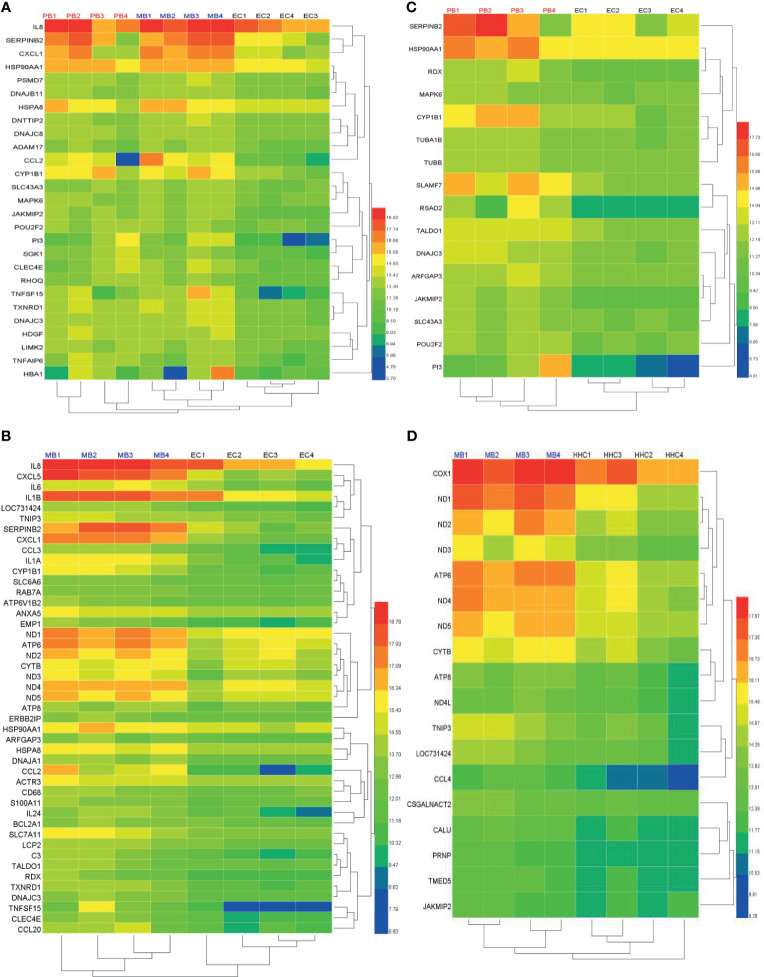
Heat map of differentially expressed genes (DEGs) in leprosy patients compared to non-leprosy controls. **(A)** The top 27 DEGs upregulated in leprosy patients (MB, n = 4; PB, n = 4) compared to ECs (n = 4). **(B)** The top 45 DEGs upregulated in MB leprosy patients (n = 4) compared to ECs (n = 4). **(C)** The top 16 DEGs upregulated in PB leprosy patients (n = 4) compared to ECs (n = 4). **(D)** The top 18 DEGs were upregulated in MB leprosy patients (n = 4) compared to HHCs (n = 4). Selection criteria were p < 0.05 and fold change ≥ 2. MB, multibacillary; PB, paucibacillary; HHCs, healthy house contacts; ECs, endemic controls.

**Figure 5:**
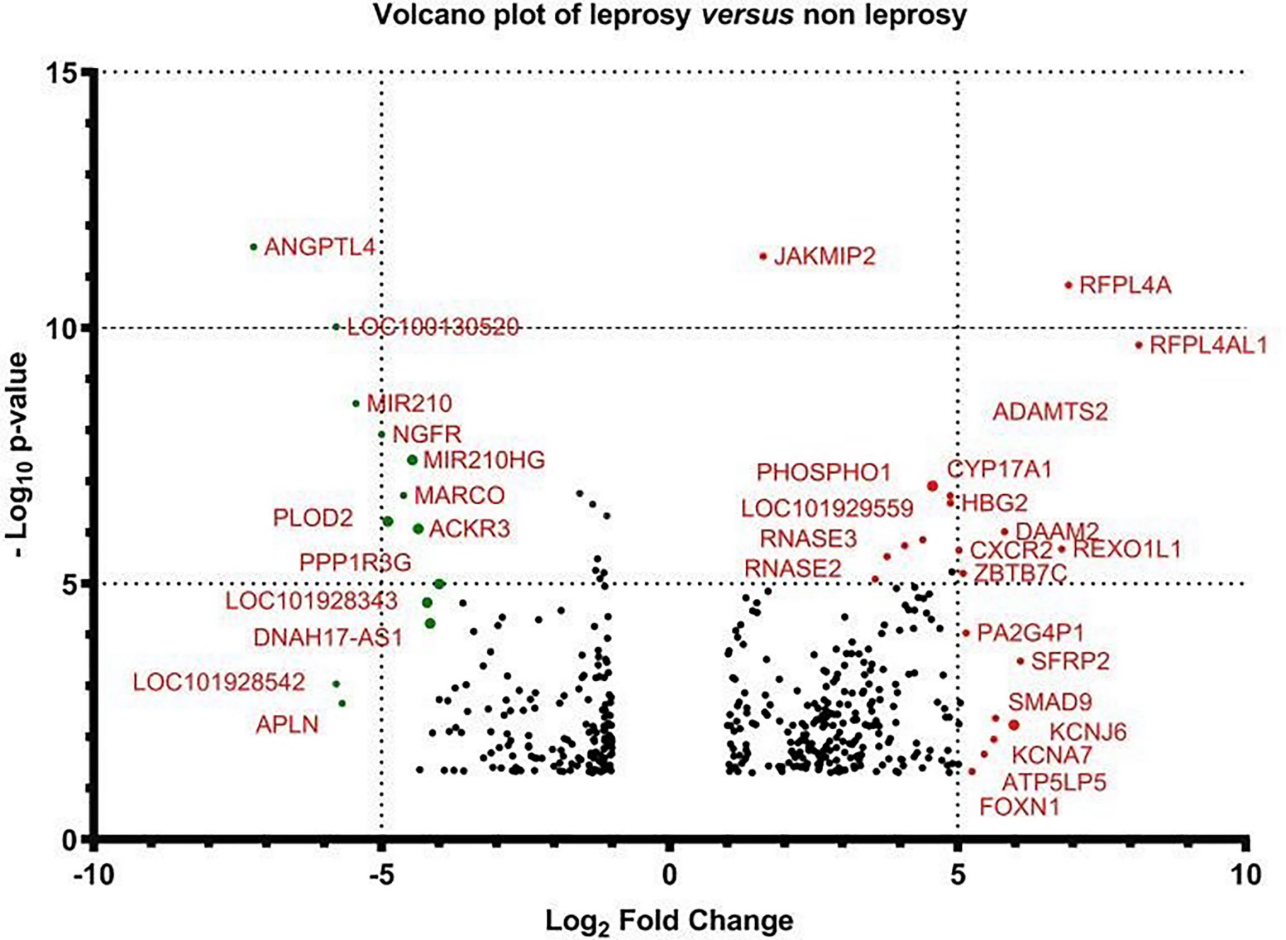
Volcano plot of the gene expression of leprosy patients versus non-leprosy controls. The logarithmic fold changes of individual genes (x-axis) are plotted against the negative log base 10 of their p-value (y-axis). Positive log_2_ (fold change) values represent upregulation in leprosy patients compared to non-leprosy controls, whereas negative values represent downregulation. Circles above the dotted line represent differentially expressed genes between leprosy patients and controls with p < 0.05 after correction for multiple testing. Red indicates upregulation and green indicates downregulation of gene expression.

We selected the top DEGs using Venn diagram analysis shown as a heat map in **Figure 4**. We found that the top 27 DEGs were upregulated in leprosy patients compared to ECs (**Figure 4A**), the top 45 DEGs were upregulated in MB leprosy patients compared to ECs (**Figure 4B**), and the top 16 DEGs were upregulated in PB leprosy patients compared to ECs (**Figure 4C**), and the top 18 DEGs were upregulated in MB leprosy patients compared to HHCs (**Figure 4D**). More details are shown in **Supplementary Table S2** and **Figure 5**.

### Performance of Selected DEGs in Distinguishing Leprosy Patients and Controls in the Validation Cohort

**Figure 6:**
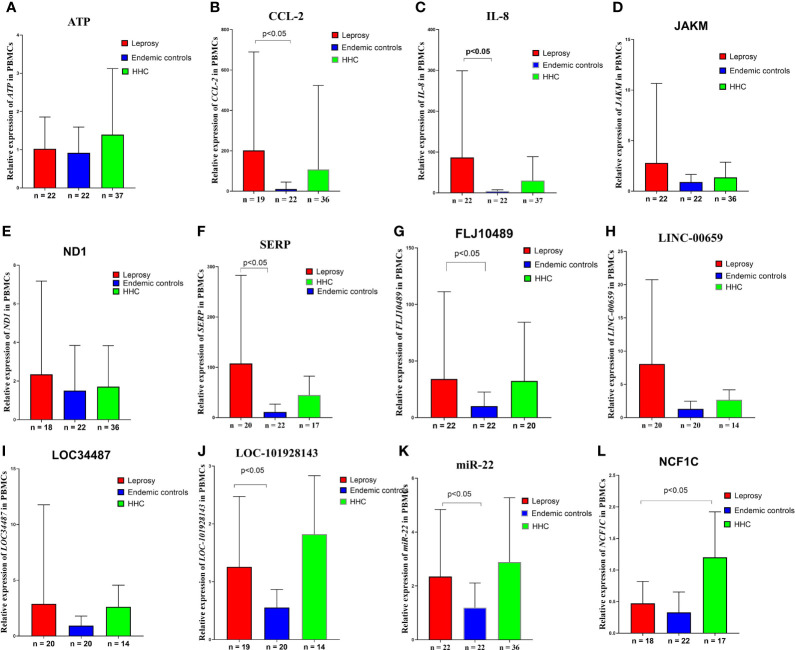
Gene expression levels in the *M. leprae*-stimulated PBMCs in the validation cohort. Differences in *ATP*, *CCL2/MCP-1*, *IL-8*, *JAKM*, *ND1*, *SERP*, *FLJ10489*, *LINC00659*, *LOC34487*, *LOC101928143*, *MIR22*, and *NCF1C* expression levels in **(A–L)** *M. leprae*-stimulated PBMC of leprosy patients, HHCs, and ECs. The cycle threshold (Ct) values from real-time PCR were normalized to the internal reference gene (*GAPDH*). 2 -ΔΔCT values are displayed. Kruskal–Wallis test and Mann–Whitney test for nonparametric data were used to evaluate statistical differences. MB, multibacillary; PB, paucibacillary; HHCs, healthy house contacts; ECs, endemic controls; DEG, differentially expressed gene; PBMC, peripheral blood mononuclear cell.

**Figure 7:**
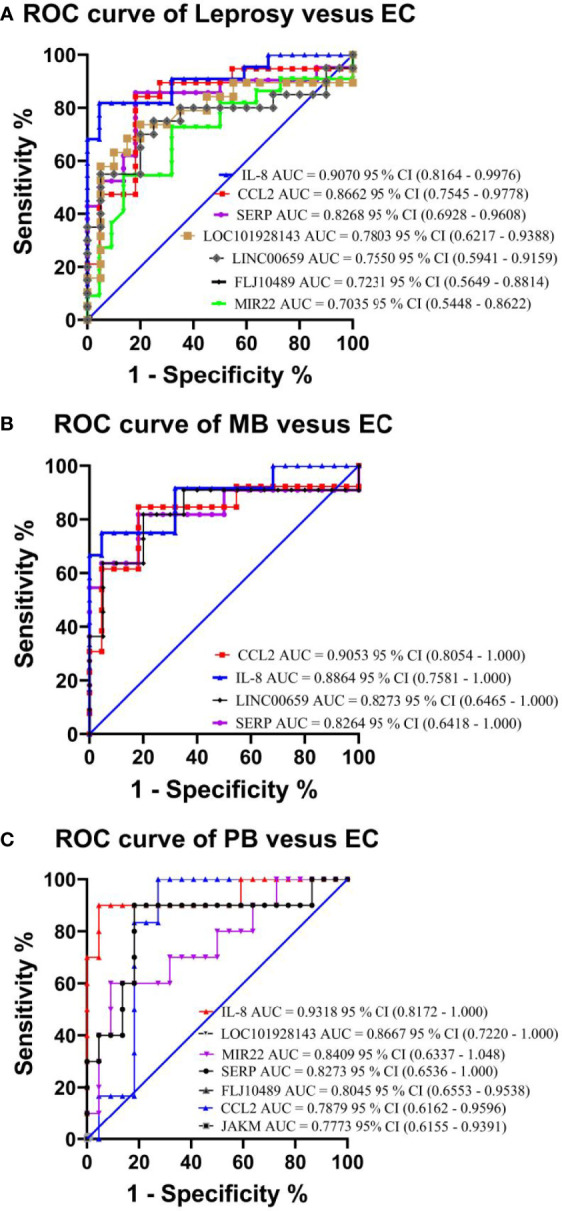
Performance test of each selected gene in the PBMCs of leprosy patients and non-leprosy controls in the validation cohort. Panels showing receiver-operating characteristic (ROC) area under the curve (AUC) for the sensitivity and specificity of the selected differentially expressed genes in RT-qPCR analysis of PBMC samples in **(A)** leprosy patients versus ECs, **(B)** MB leprosy patients versus ECs, and **(C)** PB leprosy patients versus ECs. AUC values (with 95% confidence interval) obtained by running ROC analysis are also provided. The key presents the AUC values of the selected genes. CI, confidence interval; MB, multibacillary; PB, paucibacillary; EC, endemic control.

To verify the relevance of the above identified genes, we evaluated their performance in a validation cohort, which consisted of 23 leprosy patients (13 MB and 10 PB) and 59 non-leprosy controls (37 HHCs and 22 ECs). Based on the results of RNA-seq with more than 2-fold upregulation in leprosy patients compared with non-leprosy controls followed by RT-PCR confirmation, the following 12 genes were identified and evaluated as the potential diagnostic biomarkers for leprosy: *ATP*, *CCL2/MCP-1*, *IL-8*, *JAKMIP2*, *ND1*, *SERPINB2*, *FLJ10489*, *LINC00659*, *LOC34487*, *LOC101928143*, *MIR22*, and *NCF1C*. The list of accession numbers/ID numbers for the genes as potential diagnostic biomarkers mentioned in the text and included in the NCBI search is shown in **Supplementary Table S3**. The 2 ^–ΔΔCT^ values of selected markers were determined in each participant (**Supplementary Table S4**) using the above 12 candidate genes compared between MB and PB leprosy patients and ECs or HHCs (**Table 2** and **Figure 6**). The discriminating ability of the selected signature genes was estimated using receiver-operating characteristic (ROC) curves analysis. When 23 leprosy patients were compared to 22 ECs, we found the following performance of the following candidate genes: area under the curve (AUC) of 0.9070 (95% confidence interval [CI] 0.8164–0.9976), sensitivity of 81.82%, and specificity of 95.45%, with a fold change of 18.2 for *IL-8*; AUC of 0.8662 (95% CI 0.7545–0.9778), sensitivity of 90%, and specificity of 95.45%, with a fold change of 56.8 for *CCL2*; AUC of 0.8182 (95% CI 0.6813–0.9551), sensitivity of 85.71%, and specificity of 81.82%, with a fold change of 10.5 for *SERP*; AUC of 0.7803 (95% CI 0.6217–0.9388), sensitivity of 77.3%, and specificity of 68.2% for *FLJ10489*, with a fold change of 5.7 (**Figure 7A** and **Table 2**). Furthermore, we assessed the performance of these genes in 23 leprosy patients versus 37 HHCs in the PBMCs of the validation cohort. We found that *NCF1C* had an AUC of 0.8448 (95% CI 0.7165–0.9731), sensitivity of 72.22%, and specificity of 82.35%, with a fold change of 3.8, in leprosy patients compared to HHCs (**Table 2**). In sum, *IL-8*, *CCL2*, *SERP*, and *NCF1C* showed an excellent performance in distinguishing leprosy patients from healthy controls. More details on the performance of candidate genes are shown in **Supplementary Table S4** and **Figures 7A–C**.

**Table 2:** Diagnostic potential of host biomarkers detected in *M. leprae*-stimulated PBMCs using RT-qPCR in discriminating leprosy patients from controls.

Comparison	Gene type	Gene	Fold change	P-value	AUC	95% CI	Cut-off	Sensitivity (%)	Specificity (%)
Leprosy/ECs	mRNA	*CCL2/MCP-1*	56.8	<0.0001*	0.87	0.75–0.98	>21.60	50.00	95.45
		*IL-8*	18.2	<0.0001*	0.90	0.82–0.99	>8.70	81.82	95.45
		*SERP*	10.5	0.0002*	0.83	0.69–0.96	>10.49	85.71	81.82
	lncRNA	*FLJ10489*	5.7	0.0112*	0.721	0.56–0.88	>7.13	77.27	68.18
		*LINC00659*	4.0	0.0058*	0.76	0.59–0.92	>1.62	75.00	75.00
MB leprosy/ECs	mRNA	*CCL2/MCP-1*	23.0	0.0001*	0.91	0.81–1.00	>27.35	66.67	95.45
		*IL-8*	18.2	0.0002*	0.89	0.76–1.00	>8.70	90.00	95.45
		*SERP*	9.9	0.0026*	0.83	0.64–1.00	>13.38	81.82	81.82
	lncRNA	*LINC00659*	2.2	0.0030*	0.83	0.65–1.00	>1.70	81.82	80.00
PB leprosy/ECs	mRNA	*SERP*	6.5	0.0034*	0.83	0.65–1.00	>10.49	90.00	81.82
		*IL8*	10.6	0.0001*	0.93	0.82–1.00	>9.34	90.00	95.45
		*CCL2/MCP1*	3.5	0.0334*	0.79	0.62–0.96	>3.97	66.67	81.82
	lncRNA	*FLJ10489*	2.8	0.0065*	0.80	0.66–0.95	>7.13	90.00	68.18
Leprosy/HHCs	lncRNA	*NCF1C*	3.8	0.0005*	0.84	0.72–0.97	<0.60	72.22	82.35
PB leprosy/MB leprosy	mRNA	*CCL2/MCP-1*	26.2	0.0246*	0.83	0.65–1.00	<27.97	100.00	66.67

PBMCs, peripheral blood mononuclear cells; RT-qPCR, reverse transcription quantitative polymerase chain reaction; MB, multibacillary; PB, paucibacillary; HHCs, healthy house contacts; ECs, endemic controls; mRNA, messenger RNA; lncRNA, long non-coding RNA; AUC, area under the curve; CI, confidence interval. *p < 0.05.

## Discussion

In this study, we enrolled patients with MB and PB leprosy, HHCs, and ECs. We subjected their PBMCs to *M. leprae* antigen stimulation followed by RNA-seq and identified a signature gene set (*CCL2/MCP-1*, *IL-8*, *JAKM*, *ND1*, *SERP*, *FLJ10489*, *LINC00659*, *LOC34487*, *LOC101928143*, *MIR22*, and *NCF1C*) that might potentially be useful for rapid diagnosis of leprosy. These candidate genes were then validated using PBMCs of MB and PB leprosy patients, HHCs, and ECs in a separate validation cohort, and we found that elevated expression of a 5-gene set *IL-8*, *CCL2/MCP-1*, *SERP*, *LINC00659* and *FLJ10489* could discriminate leprosy patients from ECs. In addition, increased expression of the gene set comprising *IL-8*, *CCL2/MCP-1*, *SERP* and *LINC00659* was associated with MB diagnosis, whereas upregulated expression of *IL-8*, *CCL2/MCP-1*, *SERP* and *FLJ10489* was found to be useful biomarkers for PB diagnosis. Moreover, we assessed the expression of the gene set between leprosy patients and HHCs. The results showed that the decreased expression of *NCF1C* among leprosy patients had the highest performance that could discriminate leprosy patients from HHCs. In addition, higher expression of *CCL2* among MB than PB leprosy patients could distinguish different leprosy patients. To the best of our knowledge, this is the first report on long non-coding RNA *NCF1C* that distinguished leprosy patients from healthy controls.

Studies on tuberculosis have demonstrated that IL-8 (CXCL8) is a chemokine produced mainly by macrophages and mesothelial cells (Park et al., 2003), which plays a key role in the recruitment of lymphocytes and monocytes to the pleural space in TB patients (Kurashima et al.,1997). IL-8 plays a central role in the normal immune response to *M. tuberculosis* and is required for granuloma formation (Miranda et al., 2012; Kirkaldy et al., 2003). Studies in leprosy have demonstrated that the presence of the neutrophil chemoattractant IL-8 in leprosy lesions, which do not contain neutrophils, strongly suggests a role of IL-8 as a monocyte and lymphocyte recruiter in these lesions (Park et al., 2003). IL-8 production is significantly increased in the PBMCs from patients with erythema nodosum leprosum *in vitro* when compared to those of lepromatous leprosy controls (Negera et al., 2018). Our previous study with Luminex technology has also found that IL-8 cytokine expression is elevated in leprosy patients upon stimulation with *M. leprae* antigen ML2044 (Chen et al., 2019), which confirms our findings at the transcriptomic level in this study. Thus, our finding of increased *IL-8* mRNA/gene expression in the blood from leprosy patients is consistent with a potential role for this chemokine in the pathogenesis of erythema nodosum leprosum in leprosy (Negera, et al., 2018; Bhat and Vaidya, 2020).

CCL2 (C-C motif chemokine ligand 2) is a chemokine ligand involved in immune regulation and inflammatory responses (Fang et al., 2015). The signaling pathways involved in regulating *CCL2* gene expression include cytokine receptor, NOD-like receptor, and chemokine receptor pathways. CCL2 is specifically expressed on the surface of monocytes but not on that of neutrophils and eosinophils, and its expression is associated with autoimmune diseases such as psoriasis and rheumatoid diseases (Kabala et al., 2020). Studies have shown that the 2518G/A polymorphism of *CCL2* is associated with tuberculosis and pancreatitis (Chen et al., 2015; Fang et al., 2015). Our study shows that elevated expression of *CCL2* in leprosy patients and this feature can be used to distinguish leprosy patients from non-leprosy controls. The elevated expression of CCL2 may indicate it is likely to be an inflammatory cytokine involved in the pathogenesis of leprosy. Neutrophil cytosolic factor-1C (NCF1C) is a component of NADPH oxidase, which catalyses the production of microbicidal superoxide in phagocytes and plays a vital role in host defense against microbial pathogens. The *NCF1* gene colocalizes with two pseudogenes (*NCF1B* and *NCF1C*), where *NCF1C* expression responded robustly to PMA induction during macrophage differentiation (Bakry et al., 2021; Brunson et al., 2010). A recent study from England and India indicated *NCF1C* can be an immune biomarker to differentiate active tuberculosis and latent tuberculosis (Perumal et al., 2020). Our study has shown that the expression of *NCF1C* was 3.4-fold higher among household healthy controls than that of leprosy patients (**Table 2** and **Figure 6**). This may imply that the expression of *NCF1C* may protect the close contacts of active leprosy patients from being infected with *M. leprae*, which may serve as a potential biomarker of latent infection with *M. leprae*. SERP is a serine protease inhibitor, termed serpins, which is a blood fibrinolytic inhibitor secreted in human epithelial cells to promote inflammatory responses and is a regulator of Th1 and Th2 immune cells (Boncela et al., 2013). Our result indicates that the expression of *SERP* among PMBCs stimulated by *M. leprae* antigens from leprosy patients was 10.4-fold higher than that of endemic healthy controls (**Table 2** and **Figure 6**). This may indicate that SERP may be involved in regulation of immune balance during the pathogenesis of leprosy. The functions of the other two LncRNAs including *LINC00659* and *FLJ10489* identified by our study have not yet been clarified, and their potential in the early diagnosis of leprosy is reported for the first time.

Early diagnosis is key to controlling leprosy. Currently, the gold standard diagnostic test for leprosy is based on acid-fast bacilli staining and histopathologic examination of skin lesion biopsy. *M. leprae* could not be cultured *in vitro*, and acid-fast staining requires a large number of bacilli in skin biopsy, which presents low sensibility and poor specificity (Mohanty et al., 2020). Serological tests, such as the NDO-LID Rapid Test and *M. leprae* antigen-specific ELISA are useful tools to assist in the identification of leprosy patients, especially MB leprosy patients, and the former test has a higher sensitivity but lower specificity than the latter (Chen et al., 2018). Some new methods have been developed, such as immunological assays. For example, *M. leprae* antigen-specific IFN-γ release assessed using whole-blood assay, which measures the production of IFN-γ by whole-blood specimens after co-culture with specific *M. leprae* antigens, has diagnostic value for distinguishing PB from TB but not for distinguishing PB from HHCs or ECs in the population of the southwest of China (Chen, et al., 2018). Therefore, screening novel *M. leprae*-specific antigens, combining different *M. leprae* antigens, and a multi-cytokine analytic model is needed for a more effective diagnosis of leprosy. Many studies have identified signature genes or proteins using whole-blood assay and PBMCs, which could distinguish between leprosy patients, ECs, and HHCs (Sampaio et al., 2011; Geluk et al., 2012; Oliveira et al., 2014; Freitas et al., 2016; Hungria et al., 2017; Chen et al., 2018; Chen et al., 2019). However, the relationship between transcriptome features useful for early diagnosis of leprosy is seldom discussed (Tió-Coma et al., 2019). A similar study in Vietnamese leprosy patients showed upregulation of IFN-*γ* pathway genes including IFN-*γ*, STAT1, IRF8 and IL-12 after stimulation of PBMCs by sonicated antigens (Manry et al., 2017). However, our results did not find expression of these genes to be associated with leprosy in our patients, which may be due to different ethnic groups or disease states.

Recently, a combination of four microRNAs (miR-101, miR-196b, miR-27b, and miR-29c) was found to be able to distinguish between healthy controls and leprosy patients with 80% sensitivity and 91% specificity (Jorge et al., 2017). This set of microRNAs could also discriminate between lepromatous and tuberculoid leprosy patients with a sensitivity of 83% and 80% specificity, which may have good diagnostic potential for leprosy (Jorge et al., 2017). In this study, using a different approach of transcriptomic analysis of PBMCs, we found that *IL-8*, *CCL2*, and *SERP* could not only discriminate between leprosy patients and ECs but also between MB and PB leprosy patients and ECs. In addition, *LOC34487*, *LOC101928143*, *CCL2*, and *NCF1C* had the potential to distinguish MB and PB leprosy patients from HHCs. This result is consistent with those of our previous study in a population from Southwest China, where *M. leprae* antigen (ML2044)-induced *IL-8* could distinguish PB leprosy patients from ECs in a leprosy-endemic area (Bobosha, et al., 2014; Chen et al., 2019). We also performed receiver-operating characteristic analysis using the predicted probability value in the validation cohort, and the results demonstrated the potential diagnostic value of *IL-8* and *SERP* for distinguishing leprosy patients and leprosy sub-types from ECs, and that of *NCF1C* for distinguishing leprosy patients and leprosy sub-types from HHCs.

There are some limitations in our work. First, this is a single-centre, retrospective study with a small number of patients. Second, the molecular mechanisms by which host biomarkers, such as IL-8, CCL2 regulate immune response and leprosy pathogenesis are not clear and require further clarification. Third, we only validated some mRNA and long non-coding RNA levels in this study. Several other mRNAs, such as *CXCR2*, which was also differentially expressed in the PBMCs of leprosy patients, are not validated. Genome-wide microarray or RNA-seq analysis may be the ideal way to identify microRNAs and circular RNAs with diagnostic potential, and a blood-based biomarker panel could help to improve the sensitivity and specificity of new diagnostic tests. A rapid diagnostic test based on real-time PCR analysis of the identified candidate genes for early diagnosis of leprosy could be explored in the future.

## Data Availability Statement

The datasets presented in this study can be found in NCBI online repositories. The name of the repositories and accession number can be found below: GSE179987.

## Ethics Statement

The studies involving human participants were reviewed and approved by the Medical Ethics Committee of Beijing Friendship Hospital, Capital Medical University, Beijing, P.R. Chin. Written informed consent to participate in this study was provided by the participants’ legal guardian/next of kin.

## Author Contributions

YZ and X-HC designed the study. X-HC and Y-HY prepared the manuscript. Y-HY, JL, and Y-GY conducted experiments and analysed the data. LY and YW collected and interpreted the laboratory and clinical data. YZ, YW, HL, and LY were involved in project management and organizational work. YZ, X-HC, and Y-HY reviewed the manuscript. All authors contributed to the article and approved the submitted version.

## Funding

This work was supported by the Henan Provincial Key Programs in Science and Technology (202102310355) and the Joint Program of Medical Science and Technology Research of Henan Province (SB201903018 and LHGJ20190611).

## Conflict of Interest

The authors declare that the research was conducted in the absence of any commercial or financial relationships that could be construed as a potential conflict of interest.

## Publisher’s Note

All claims expressed in this article are solely those of the authors and do not necessarily represent those of their affiliated organizations, or those of the publisher, the editors and the reviewers. Any product that may be evaluated in this article, or claim that may be made by its manufacturer, is not guaranteed or endorsed by the publisher.

## Abbreviations

AUC, area under the curve; CI, confidence interval; DEG, differentially expressed gene; EC, endemic control; HHC, healthy house contact; MB, multibacillary; PB, paucibacillary; PBMC, peripheral blood mononuclear cell; RIN, RNA integrity number; RNA-seq, RNA sequencing.
