# Supplementary material for: Transcriptomic Analysis of Mycobacterium leprae-Stimulated Response in Peripheral Blood Mononuclear Cells Reveal Potential Biomarkers for Early Diagnosis of Leprosy
Source: Front Cell Infect Microbiol. 2021 Dec 21;11:714396. doi: 10.3389/fcimb.2021.714396 (PMC8724050; doi:10.3389/fcimb.2021.714396)
Supplement: Supplementary file 1 [file DataSheet_1.doc]

Supplementary Material

# Supplementary Tables

**Supplementary Table S1**. Primers of the selected differentially expressed genes and *GAPDH* for RT-qPCR.

| **Gene** | **Gene type** | **Position** | **Primer sequence (5’-3’)** |
| --- | --- | --- | --- |
| *ATP6* | ncRNA | Forward | CTTATGAGCGGGCACAGTGA |
|  |  | Reverse | GATATTGCTAGGGTGGCGCT |
| *CCL2/MCP1* | mRNA | Forward | ATAGCAGCCACCTTCATTCC |
|  |  | Reverse | ATCCTGAACCCACTTCTGCT |
| *COX1* | mRNA | Forward | TCCCCTAATAATCGGTGCCC |
|  |  | Reverse | GTTAGGTCTACGGAGGCTCC |
| *FLJ10489* | miscRNA | Forward | ACAGATGGCCTGGATCAGTG |
|  |  | Reverse | TCCAAAAGCTTCCACTACCTCC |
| *HAVCR2* | mRNA | Forward | TGTGCCTAACAGAGGTGTCC |
|  |  | Reverse | TTCCACTTCTGAGGACCTTGT |
| *IL8*  *JAKMIP2*  *LINC00659*  *LOC100419706*  *LOC101928143*  *LOC34487*  *MIAT*  *MIR22HG*  *MT-ND1P23*  *NCF1C*  *ND1*  *ND2*  *ND3*  *PRNP*  *SERPINB2*  *THBS1*  *CXCR2*  *CXCR2*  *GAPDH* | mRNA | Forward | GCTCTGTGTGAAGGTGCAGTT |
|  |  | Reverse | TTTCTGTGTTGGCGCAGTGT |
| *JAKMIP2*  *LINC00659* | mRNA | Forward | ACGAAGTCAAAGGACCCGAG |
|  |  | Reverse | TCTCTGGCCCATGTTCCCTA |
| *LINC00659* | miscRNA | Forward | AGGATGTCAGCCTCTGCTTG |
|  |  | Reverse | GTGTGCACAACCTCAAAGCC |
| *LOC100419706* | ncRNA | Forward | TCCAGAAGTCAGAGCTTGCT |
|  |  | Reverse | TAACTCACTGGGAAGGGCTC |
| *LOC101928143* | lncRNA | Forward | CTTGTTCCAGCGTGGAAAGC |
|  |  | Reverse | GGCAAAAGCACTGGCTAAACA |
| *LOC34487* | lncRNA | Forward | ATGGAAGACGTGGGCTTGAG |
|  |  | Reverse | AGCTTCCGGGGTAATCTCCT |
| *MIAT* | miscRNA | Forward | GTGCCTGGGAAGTGGTAGAG |
|  |  | Reverse | GAGGGTCGGGTATCATCTCA |
| *MIR22HG* | miscRNA | Forward | TTTCTGCCTCCGAGATGTGG |
|  |  | Reverse | TGGTCCTTGAGGCTGCTTAT |
| *MT-ND1P23* | mRNA | Forward | ACGGGCTACTACAACCCTTC |
|  |  | Reverse | GCCTAGGTTGAGGTTGACCA |
| *NCF1C* | lncRNA | Forward | CTGGAGGCCACCCAGTCAT |
|  |  | Reverse | CACCAGGAACATGTACATAGTGC |
| *ND1* | mRNA | Forward | GCCACATCTACCATCACCCT |
|  |  | Reverse | ATGCTCACCCTGATCAGAGG |
| *ND2* | mRNA | Forward | ATCTCGCACCTGAAACAAGC |
|  |  | Reverse | GCTATGATGGTGGGGATGA |
| *ND3* | mRNA | Forward | ACCACAACTCAACGGCTACA |
|  |  | Reverse | GTAGGGGTAAAAGGAGGGCA |
| *PRNP* | mRNA | Forward | GTGCACGACTGCGTCAAT |
|  |  | Reverse | CCTTCCTCATCCCACTATCAGG |
| *SERPINB2* | mRNA | Forward | GCATGTTCTTGTTGCTTCCA |
|  |  | Reverse | TTCAGCCATTTTGTCTTTGC |
| *THBS1* | mRNA | Forward | TTCCACTTCTGAGGACCTTGT |
|  |  | Reverse | TGCTTATTTGTTCTCTACTGGCT |
| *CXCR2* | mRNA | Forward | TGGGTACAGTGCTATTCTGCC |
|  |  | Reverse | TAAATCCTGACTGGGTCGCTG |
| *GAPDH* | mRNA | Forward | CCCCTTCATTGACCTCAACTAC |
|  |  | Reverse | GATGACAAGCTTCCCGTTCTC |

RT-qPCR, reverse transcription quantitative polymerase chain reaction; mRNA, messenger RNA; lncRNA, long non-coding RNA; ncRNA, non-coding RNA; miscRNA, miscellaneous RNA.

**Supplementary Table S2.** List of differentially expressed genes validated by RT-PCR in leprosy patients versus controls.

| **Gene** | **Gene name** | **Gene type** | **NCBI gene ID** |
| --- | --- | --- | --- |
| *ATP* | MT-ATP6 (mitochondrially encoded ATP synthase 6) | ncRNA | 4508 |
| *CCL2/MCP1* | CCL2 (C-C motif chemokine ligand 2) | mRNA | 6347 |
| *FLJ10489* | LINC01181 (long intergenic non-protein coding RNA 1181) | miscRNA | 379034 |
| *IL8* | CXCL8 (C-X-C motif chemokine ligand 8) | mRNA | 3576 |
| *JAKMIP2* | JAKMIP2 (janus kinase and microtubule interacting protein 2) | mRNA | 9832 |
| *LINC00659* | LINC00659 (long intergenic non-protein coding RNA 659) | miscRNA | 100652730 |
| *LOC101928143* | LOC101928143 (uncharacterised LOC101928143) | miscRNA | 101928143 |
| *ND1* | NADH-ubiquinone oxidoreductase chain 1 | ncRNA | 4535 |
| *LOC344887* | NMRAL2P (NmrA-like redox sensor 2, pseudogene) | miscRNA | 344887 |
| *MIR22HG* | MIR22HG (MIR22 host gene) | miscRNA | 84981 |
| *NCF1C* | NCF1C (neutrophil cytosolic factor 1C pseudogene) | miscRNA | 654817 |
| *SERPINB2* | SERPINB2 (serpin family B member 2) | mRNA | 5055 |

NCBI, National Center for Biotechnology Information; ncRNA, non-coding RNA; mRNA, messenger RNA; miscRNA, miscellaneous RNA.

**Supplementary Table S3.** Overall DEGs showing a significantly altered expression in leprosy patients compared to non-leprosy controls.

| **Leprosy patients versus**  **non-leprosy controls** | | **DEGs (n)** | | | | |
| --- | --- | --- | --- | --- | --- | --- |
|  |  | **All (n)** | **Upregulated(n)** | **Fold** | **Downregulated (n)** | **Fold** |
| Leprosy | versus (HHCs+ECs) | 423 | 260 | 2.02–282.44 | 163 | 0.01–0.50 |
| MB |  | 723 | 272 | 2.01–154.07 | 451 | 0.00–0.50 |
| PB |  | 314 | 232 | 2.04–410.82 | 82 | 0.00–0.50 |
| Leprosy | versus *ECs* | 1853 | 918 | 2.00–433.80 | 935 | 0.00–0.49 |
| MB |  | 2395 | 1382 | 2.00–753.15 | 1013 | 0.00–0.49 |
| PB |  | 1582 | 741 | 2.00–608.19 | 841 | 0.00–0.50 |
| Leprosy | versus *HHCs* | 69 | 48 | 2.01–141.22 | 21 | 0.05–0.50 |
| MB |  | 389 | 155 | 2.00–77.03 | 234 | 0.00–0.50 |
| PB |  | 70 | 52 | 2.28–205.41 | 26 | 0.00–0.49 |
| MB versus PB | | 203 | 82 | 2.05–8280.36 | 121 | 0.00–0.50 |
| HHCs versus ECs | | 1333 | 592 | 2.00–133.60 | 741 | 0.00–0.50 |

DEGs, differentially expressed genes; MB, multibacillary; PB, paucibacillary; HHCs, healthy house contacts; ECs, endemic controls.

**Supplementary Table S4.** Median and IQR values of host markers detected in the RT-qPCR analysis of *M. leprae*-stimulated PBMCs in leprosy (MB and PB) patients and non-leprosy controls (ECs and HHCs).

| **Gene type** | **Host marker** | **Leprosy patients (median, IQR)** | **MB leprosy patients (median, IQR)** | **PB leprosy patients (median, IQR)** | **HHCs (median, IQR)** | **ECs (median, IQR)** |
| --- | --- | --- | --- | --- | --- | --- |
| mRNA | ATP | 0.91 (0.43–1.43) | 0.89 (0.49–1.24) | 1.05 (0.27–1.64) | 0.78 (0.34–1.83) | 0.77 (0.45–1.18) |
|  | Number of values | 22 | 12 | 10 | 37 | 22 |
|  | CCL2/MCP-1 | 14.00 (5.2–50.02) | 43.79 (5.93–225.7) | 6.28 (3.58–16.04) | 12.96 (2.12–45.3) | 0.69 (0.29–2.05) |
|  | Number of values | 19 | 13 | 6 | 37 | 22 |
|  | IL-8 | 13.89 (9.91–45.0) | 23.64 (7.25–57.7) | 13.75 (10.9–32.2) | 7.35 (1.76–34.1) | 2.31 (1.52–7.17) |
|  | Number of values | 22 | 12 | 10 | 37 | 22 |
|  | JAKM | 0.79(0.28–1.39) | 0.39 (0.19–0.95) | 1.19 (0.77–3.38) | 0.83 (0.54–1.47) | 0.70 (0.24–1.02) |
|  | Number of values | 22 | 12 | 10 | 36 | 22 |
|  | MTND1 | 0.89 (0.28–2.49) | 0.83 (0.4–3.17) | 1.19 (0.09–6.72) | 0.74 (0.39–2.3) | 0.79 (0.41–1.32) |
|  | Number of values | 18 | 12 | 6 | 36 | 22 |
|  | SERP | 52.46 (16.9–122.6) | 96.93 (16.5–123.3) | 28.64 (20.9–138.2) | 33.44 (25.0–54.2) | 4.12 (1.64–10.02) |
|  | Number of values | 21 | 11 | 10 | 17 | 22 |
| lncRNA | FLJ10489 | 14.58 (7.14–22.3) | 14.68 (3.19–27.12) | 14.10 (7.5–22.49) | 14.17 (2.2–32.3) | 5.06 (2.3–9.59) |
|  | Number of values | 22 | 12 | 10 | 20 | 22 |
|  | LINC00659 | 2.33 (1.48–9.83) | 2.39(1.76–10.41) | 1.90 (0.5–8.47) | 2.48 (1.4–3.43) | 1.09 (0.54–1.63) |
|  | Number of values | 20 | 11 | 9 | 14 | 20 |
|  | LOC34487 | 1.02 (0.34–1.72) | 1.10 (0.2–1.82) | 1.02 (0.44–1.59) | 1.78 (1.0–4.35) | 0.63(0.26–0.96) |
|  | Number of values | 21 | 12 | 9 | 14 | 20 |
|  | LOC101928143 | 1.09 (0.67–1.34) | 1.03 (0.4–2.06) | 1.14 (0.83–1.31) | 1.490(0.96–2.74) | 0.49 (0.35–0.71) |
|  | Number of values | 19 | 10 | 9 | 14 | 20 |
|  | MIR22 | 1.87 (0.99–2.74) | 1.53 (1.01–2.15) | 2.35 (0.96–3.52) | 2.160(1.49–3.46) | 0.97 (0.63–1.54) |
|  | Number of values | 22 | 12 | 10 | 36 | 22 |
|  | NCF1C | 0.48(0.12–0.69) | 0.42(0.09–0.59) | 0.53 (0.16–0.88) | 1.01 (0.68–1.53) | 0.23(0.16–0.35) |
|  | Number of values | 18 | 8 | 10 | 17 | 22 |

mRNA, messenger RNA; lncRNA, long non-coding RNA; MB, multibacillary; PB, paucibacillary; HHCs, healthy house contacts; ECs, endemic controls; IQR, interquartile range.
